# Historical Perspective of the Characterization of Conotoxins Targeting Voltage-Gated Sodium Channels

**DOI:** 10.3390/md21040209

**Published:** 2023-03-27

**Authors:** James R. Groome

**Affiliations:** Department of Biological Sciences, Idaho State University, Pocatello, ID 83209, USA; groojame@isu.edu

**Keywords:** conotoxin, sodium channel, voltage-gated, pore blocker, gating modifier

## Abstract

Marine toxins have potent actions on diverse sodium ion channels regulated by transmembrane voltage (voltage-gated ion channels) or by neurotransmitters (nicotinic acetylcholine receptor channels). Studies of these toxins have focused on varied aspects of venom peptides ranging from evolutionary relationships of predator and prey, biological actions on excitable tissues, potential application as pharmacological intervention in disease therapy, and as part of multiple experimental approaches towards an understanding of the atomistic characterization of ion channel structure. This review examines the historical perspective of the study of conotoxin peptides active on sodium channels gated by transmembrane voltage, which has led to recent advances in ion channel research made possible with the exploitation of the diversity of these marine toxins.

## 1. An Overview of Bioactive Marine Toxins

A great diversity of marine organisms produce and secrete bioactive toxins, which have apparently evolved as an evolutionary strategy for increasing fitness by enhancing functions ranging from predictable defense against predation, prey capture, and immobilization [1,2]. As marine and other bioactive toxins have been identified and their effects partially or fully characterized, additional research has focused on evolutionary patterns of development of resistance to these toxins [3,4,5,6,7]. The venom of marine cone snails (*Conus* sp.) has attracted considerable research attention with studies of behavioral strategies, evolution, and toxicity [8,9,10,11,12,13]. A large diversity of species in this molluscan genus produce a plethora of bioactive conopeptides, with over 700 species and more than 10,000 conotoxin peptides identified to date [14]; for review of conotoxin gene families see [15], noting that many additional conotoxin genes have been sequenced [16]. This research has been motivated in part as an element of evolutionary studies as mentioned above, and also by an interest in determining sequence- and disulfide-linkage determinants of toxin binding and isoform-specific bioactivity. Functional characterizations of conopeptides and conotoxins have provided unique opportunities to study ligand-gated receptor channels, voltage-gated channels, transport proteins, and GTP binding proteins in specific tissues (for reviews see [17,18,19,20,21,22]).

In addition, these toxins have been invaluable as part of structural studies (reviewed by [23,24,25]) and provide promise for pharmacological intervention in disease (reviewed by [26,27,28,29,30,31,32,33]). Progress in the development of conopeptides or analogues as analgesics has resulted in FDA approval of Ziconotide, or synthetic ω-conotoxin MVIIA [34]. Investigation of the therapeutic potential of conopeptides for nonopioid analgesia continues (reviewed by [35,36,37]), and a recent study demonstrating agonist activity of *Conus* venom extracts on CB1 receptors as determined by internalization and resulting in significant analgesic or antinociceptive effects in mice [38]. Acceleration of the development of conopeptide analogues may be facilitated with alternative synthesis approaches as has been shown for the μ-conotoxin KIIIA [39].

Many α-conotoxins have been purified or synthesized, and their actions on nicotinic acetylcholine receptors (nAChRs) have been and continue to be investigated (for reviews see [40,41,42,43,44,45,46]). The diversity of α-conotoxins has been exploited to investigate acetylcholine receptors comprising subunit combinations that are determinants of synaptic communication in the nervous system and periphery (reviewed by [47]). This work has evolved to include robust computational approaches including molecular dynamics and in silico scanning of potential α-conotoxin analogues (reviewed by [48]); recent work includes [49,50,51,52,53,54]. These and functional investigations have explored α-conotoxin specificity in the characterization of nAChR permutations, and the potential therapeutic use of naturally occurring, synthetic, and mutant α-conotoxins to treat neurodegenerative diseases including Parkinson’s Disease [55,56,57,58,59,60], as well as management of peripheral painful neuropathies [51,61,62,63,64,65,66].

## 2. Toxins Targeting Voltage-Gated Sodium Channels

**Figure 1:**
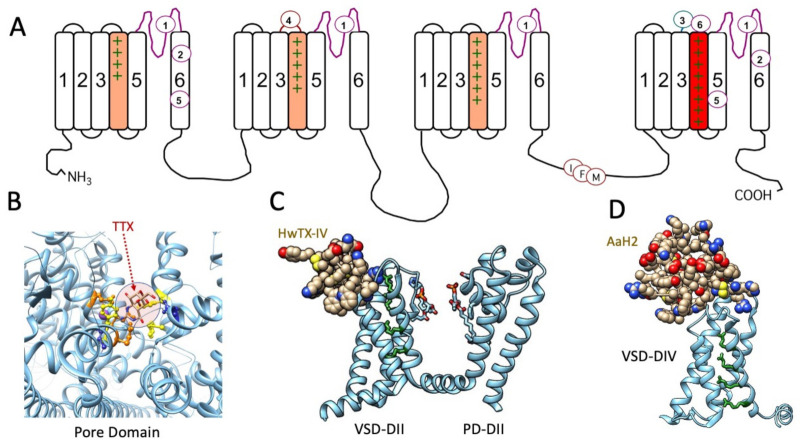
(**A**) Diagram of the sequence of the voltage-gated sodium channel, with voltage sensor S4 segments shaded in domains I–III (orange) and in domain IV (red) to denote the role of DIV S4 (domain IV S4 segment) in fast inactivation, with the inactivation particle identified as the IFM motif in the DIII–DIV linker. Receptor sites for marine toxins and extending to that for veratridine/batrachotoxin (site 2) are indicated, excluding insecticide and other receptor binding sites. Cryo-EM structures of toxins bound to sodium channels are shown below. (**B**) TTX bound within receptor site 1 in the pore domain of hNa_V_1.4, with the toxin shaded (arrow). Binding site loci include acidic residues glutamate (orange), aspartate (blue), and aromatic residues (yellow): .pdb 6A95 [82]. (**C**) Spider toxin HwTX-IV bound to receptor site 4 in the extracellular S3–S4 linker of the voltage sensor domain (VSD) of domain II in hNa_V_1.7, with DII pore domain (PD) included in this view: .pdb 7K48 [83]. (**D**) α-scorpoin toxin AaH2 bound to receptor site 3 in the extracellular S3–S4 linker of the voltage sensor domain of domain IV of hNa_V_1.7: .pdb 6NT4 [84]. S4 positively charged residues are shown in green for (**A**,**C**,**D**) and illustrate the activated position of DIIS4 with HwTX-IV bound (**C**) and resting position of DIVS4 with AaH2 bound (**D**).

Biological toxins with potent actions on voltage-gated sodium channels act on numerous receptor sites (reviewed by [67]; Figure 1A). Alkaloid guanidinium toxins such as saxitoxin (STX), tetrodotoxin (TTX), and the μ-conotoxins act at site 1 within the outer vestibule of the pore module, to limit sodium permeation during channel opening. Receptor site 2 in S6 of domain I and domain IV, and perhaps extending to domains II and III [68,69], bind plant alkaloids such as veratridine and animal toxins such as batrachotoxin. Other bioactive toxins including dinoflagellate ciguatoxins and brevetoxins act at site 5 in DI S6 and involve DIV S5 (reviewed by [17,70,71,72,73]) to promote activation and slow the entry of sodium channels into the fast inactivated state. Sea anemone, spider, and scorpion toxins bind to extracellular loops comprising receptor sites 3 or 4 in the voltage-sensing module of domains IV and II, respectively. These toxins modify channel gating by trapping the S4 segment voltage sensor in resting or activated states (reviewed by [22,74,75,76,77,78,79]). Conotoxins target sites in each of these S3–S4 extracellular loops. For example, receptor site 6 is, similar to site 3, in the domain IV S3–S4 linker and is targeted by δ-conotoxins that slow the entry of channels into a state of fast inactivation [80]. The μO-conotoxins have actions suggesting they act, in part, at receptor site 4 to limit sodium permeance, through gating modifying actions similar to β scorpion toxins [81]. A revised convention for these binding site models is provided in [67].

Cryo-EM structures of several voltage-gated sodium channels in complex with guanidinium or animal toxins have supported the functional experiments localizing toxin receptor binding sites, detailing specific interactions of toxins with binding site residues, and confirming toxin actions as pore blockers or gating modifiers. A few of these toxin-bound channel structures are illustrated in Figure 1: tetrodotoxin (TTX) in receptor site 1 (Figure 1B), spider toxin in receptor site 4 (Figure 1C), and α-scorpion toxin in receptor site 3 (Figure 1D).

## 3. Conotoxins Targeting Voltage-Gated Sodium Channels

The expanding database for conopeptide gene superfamilies, sequence, structure, and biological actions is updated in the ConoServer website (http://www.conoserver.org) described in [85]. Conotoxins are typically classified according to their endoplasmic signal sequence into one of about a dozen superfamilies, and further classified by cysteine framework into a larger set of conotoxin families acting on target channels, receptors, or transporters (reviewed by [86]). The structural diversity of conotoxins acting on voltage-gated sodium channels originates from a limited set of cysteine frameworks, for which folding patterns dictated by sequence are the primary determinant of the structural diversity, as suggested by comparisons of the M and O superfamilies [87] and later extended to the remaining conotoxin gene superfamilies [86]. Structural features for conotoxins targeting voltage-gated sodium channels are shown in Table 1.

**Figure 2:**
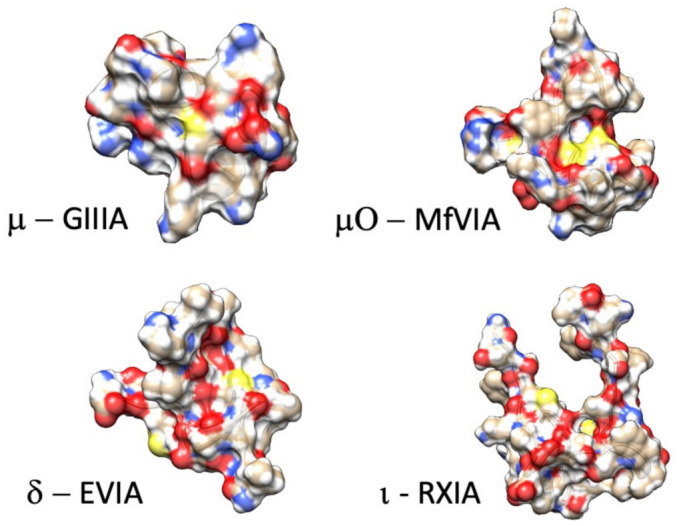
Example structures of functionally characterized conotoxins (µ-conotoxin GIIIA: .pdb 1TCK [92]; µO-conotoxin MfVIA: .pdb 2N7F [93]; δ-conotoxin EVIA: .pdb 1G1P [94]; ι-conotoxin RXIA: .pdb 2JRY [95]).

Here, we review from a historical perspective, the isolation, synthesis, and structural determination, with a principal focus on the functional characterization of the actions of conotoxins on voltage-gated sodium channels. In the M superfamily of conotoxins, these peptides act as pore blockers (µ-conotoxins), whereas in the O superfamily, µO-conotoxins prohibit sodium permeance by acting as gating modifiers (for reviews see [81,88,89,90]). Other conotoxins act as gating modifier toxins that influence fast inactivation or activation (δ-conotoxins and ι-conotoxins; for reviews see [19,91]). Example structures for each of these families of conotoxins targeting sodium channels are shown in Figure 2. As shown in Figure 1 and further discussed in this review, their binding sites are consistent with actions to block the sodium channel pore or alter voltage-dependent gating transitions. Recent investigations employing cryo-EM, homology modeling, molecular dynamics, and transcriptome approaches have enhanced our understanding of the structure-to-function relations of these conotoxins and their target sodium channel isoforms.

## 4. Isolation and Initial Characterization of Actions of μ-Conotoxins on Nerve and Muscle

Conotoxins acting on voltage-gated sodium channels were discovered several decades ago along with α- and β- scorpion venom toxins, certain alkaloids, and the guanidinium toxins tetrodotoxin and saxitoxin used in biochemical purification of sodium channels from elasmobranch electric organs and mammalian tissue. Venom extracts from *Conus geographus* were shown to inhibit electrical and contractile activity of skeletal (but not smooth or cardiac) muscle in 1974 [96]. Micro conotoxins from this species were then isolated; purified as GIIIA, GIIIB, and GIIIC; and tested functionally [97,98,99]; initial reports referred to these μ-conotoxins as geographutoxins. Their functional characterization showed their inhibition of muscle action potentials elicited either with stimulation of the motor nerve or by direct muscle stimulation. In those experiments, GIIIA applied during indirect stimulation of the muscle (via motor nerve) blocked the compound muscle action potential muscle leaving the endplate depolarization unaffected, suggesting toxin action on the sarcolemmal voltage-gated sodium channels. This was confirmed with the observation that the muscle fiber action potential was prohibited with application of GIIIA during direct stimulation of the muscle. Additional experiments in these and other studies showed that neuronal sodium channel activity (synaptosome preparations) was not blocked by GIII toxins, suggesting that μ-conotoxins specifically target skeletal muscle sodium channels [100,101,102].

The isolation of μ-conotoxins from *Conus geographus* was accomplished in the same period as that for the cloning of sodium channels from electric fish [103]. Sodium channel genes were subsequently identified in mammalian brain [104,105], and in cardiac [106,107,108] and developing [109] or adult [110,111] skeletal muscle. Functional expression of these genes in heterologous expression with messenger RNA [110,112,113,114,115,116] was typically associated with tests for the actions of STX and TTX, and often with GIIIA. These and additional results supported the hypothesis that GIIIA targeted TTX-sensitive skeletal muscle sodium channels (Na_V_1.4) with little effect on TTX-resistant sodium channels of developing skeletal muscle or the myocardium (Na_V_1.5), or sodium channels in brain (i.e., Na_V_1.2). As additional sodium channel genes from brain and peripheral nervous tissues were cloned and functionally characterized, this specificity was modified slightly as GIIIA, GIIIB, and GIIIC were shown to compete, albeit weakly, with STX in radiolabeling binding assays in brain tissue [117] and produce partial inhibition of certain neuronal sodium channels, i.e., TTX-sensitive Na_V_1.6 [118].

**Figure 3:**
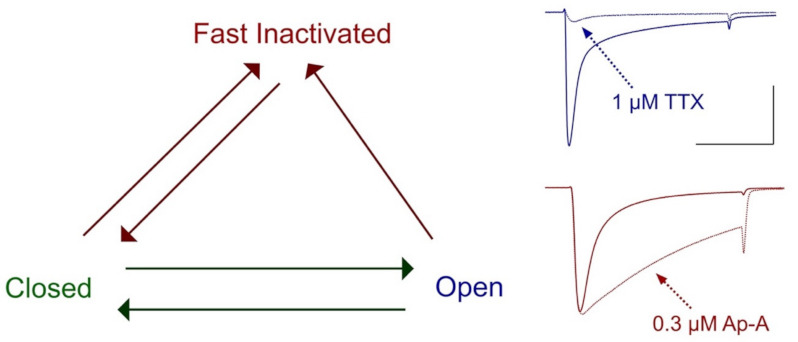
Voltage-dependent state transitions in sodium channels. Channels respond to weak depolarization by inactivating, or to strong depolarization by opening prior to inactivation. At right is shown the action of the site-1 toxin tetrodotoxin (TTX) to block sodium permeance in hNa_V_1.4 channels (dotted line, blue) and is compared to the action of the site-3 toxin Anthopleurin-A (Ap-A) to slow the entry of these channels into fast inactivation (dotted line, red). Calibration; vertical 1 μA, horizontal 10 ms. Groome, unpublished.

Importantly, cloning of voltage-gated sodium channels facilitated investigations into the mechanism of μ-conotoxin binding to the sodium channel, for which specific regions had now been associated with channel functions of activation and fast inactivation [113]. Characterization of the effects of saxitoxin and tetrodotoxin showed that these bind to receptor site 1 in the pore to block sodium entry through open channels, whereas sea anemone toxins such as the anthopleurins bind to receptor site 3 (extracellular S3–S4 loop in the domain IV voltage sensor module, impeding the translocation of the domain S4 segment, to slow the entry of sodium channels into a state of fast inactivation [119,120] and reviewed by [78] (Figure 3). Use of GIII μ-conotoxins in the aforementioned studies to characterize sodium channel genes with their functional expression showed that these μ-conotoxins acted, similar to the guanidinium toxins STX or TTX, as pore blockers. Descriptions of the initial characterization and tests on expressed sodium channels for these and additional conotoxins follows.

Since TTX binds effectively to the pore of neuronal as well as skeletal muscle sodium channels, the finding that GIIIA block was specific to skeletal muscle channels revealed differences in the site 1 receptor in these channel isoforms [121], and that the developmental stage of embryonic muscle is critical for GIIIA potency [100,101]. Experiments with radiolabeled TTX, STX, or GIIIA in competition binding assays suggested that the guanidinium toxins and μ-conotoxins share an overlapping region of binding in receptor site 1 [121,122,123,124]. This hypothesis was supported by the results of single channel recordings illustrating differential wash out under conditions of single or co-occupancy of guanidinium and GIIIA toxin and gating current recordings showing that TTX and GIIIA have direct and distinguishable actions on S4 voltage sensor translocation [125,126,127].

NMR determination of the three-dimensional structures of GIIIA [128] and GIIIB [129] revealed similar, compact structures, stabilized by three disulfide bridges, and helices orienting positively charged arginine or lysine residues into the aqueous solvent, suggesting their potential interaction of the outer vestibule region in the pore of the skeletal muscle sodium channel. Studies by [130] using rat diaphragm muscle bioassay and [131] using radiolabeling binding assay and single channel recordings showed that the guanidinium side chain of Arg-13 was essential for μ-conotoxin binding and channel occlusion, with other arginine, lysine, or hydroxyproline residues providing lesser but significant determinants of toxin binding. An NMR study of GIIIA mutations supported the notion that Arg-13 substitution did not (indirectly) inhibit binding as the result of conformational alteration of the toxin, and it also further supported roles for other arginine, lysine, and hydroxyproline residues in channel interaction and overlap with the TTX and STX binding site [92]. However, conformational effects as determined with NMR were correlated with significant decrease in toxin potency for glutamate substitution at Asp-12 [132]. Conformational perturbation with alanine substitutions at individual cysteine residues significantly decreased binding efficacy of GIIIA, for each of the three disulfide linkages [133]. The NMR determination of the structure of GIIIC [118] revealed that naturally occurring differences at position 16 between GIIIC (Leu-16) and the other μ-conotoxins affected both the conformation of this toxin and its differential block of Na_V_1.4 (skeletal muscle) versus Na_V_1.6 (brain).

**Figure 4:**
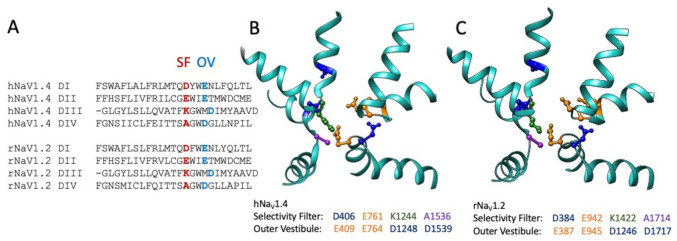
Sequence alignment (**A**) of the pore helices for sodium channels hNa_V_1.4 (**B**): .pdb 6AGF [82]) and rNa_V_1.2 (**C**): .pdb 6J8E [152]). The selectivity filter (SF) is shown in red for the alignment, with acidic residues of the outer pore vestibule (OV) shown in sky blue. Residues within the pore helical structures in (**B**,**C**) are listed as acidic aspartate and glutamate, positive lysine, and neutral alanine. Sequence alignments were performed in Seaview 5 [153].

The role of Arg-13 and other residues in GIIIA in toxin binding were assessed in several investigations collectively utilizing a variety of approaches to test for interaction with toxin and pore residues. Sodium permeation is dependent on outer vestibule acidic residues and the constricted inner pore selectivity filter ([134,135,136,137,138,139]; Figure 4). Specific skeletal muscle sodium channel pore acidic residues were suggested as the target for GIIIA Arg-13. Point mutations in the outer vestibule of the pore and extending to the selectivity filter negatively impacted GIIIA affinity [140,141,142,143]. These and additional studies suggested that binding of GIIIA involves the concerted action of multiple toxin residues interacting with outer vestibule pore residues and favoring the binding pose of Arg-13 inserted more deeply into the pore [144,145,146,147,148] and included at least one neutral toxin residue, A22 [149]. Interestingly, differences between GIIIA and GIIIB binding were correlated with naturally occurring differences in toxin residues that mediate their differential interactions with the sodium channel pore [150,151].

Thiosulfonate reagents were used with cysteine-substituting mutations to show that residue charge and structure (bulkiness of side chains as for tryptophan) contribute to GIIIA binding in the pore region [142,154] and furthered our understanding of the anionic “DEKA” selectivity filter [148,155,156] and of slow inactivation [157]. These mutant cycle analyses of several GIIIA or GIIIB Arg-13 mutants, sodium channel pore mutants including but not restricted to the selectivity filter, and their respective double toxin/channel mutational perturbation were used to calculate free energy (differences) for toxin interaction with the pore. These studies also identified electrostatic and other interactions of the toxin with outer pore vestibule residues outside of the selectivity filter, and they elucidated the tilted orientation of GIIIA as it bound to the pore. Functional characterizations of the effects of mutations of negatively charged residues in the outer pore ring on GIIIA binding were used to direct molecular dynamics simulations that largely confirmed the emerging consensus for μ-conotoxin binding in the outer vestibule of the pore module, and with its relative bulkiness, binding less deeply in the pore than STX or TTX [146]. 

An additional, interesting result from investigation of the binding of GIIIA to the skeletal muscle sodium channel pore was the determination of the organization of domains in this channel [158]. The clockwise orientation of hNa_V_1.4 domains is of considerable relevance in investigations of sodium channel gating for which perturbations in voltage sensor or pore domains may directly or allosterically influence function in their counterpart domain, as many voltage-gated sodium channels exhibit pairwise, swapped domains (i.e., the pore module in DI is closest to the voltage sensor module in DIV in hNa_V_1.4). Most importantly, these studies gave an early view of the structure of the sodium channel pore, in addition to an understanding of μ-conotoxin action.

## 5. Isolation and Characterization of μ-Conotoxins Targeting Neuronal Sodium Channels

Following the isolation of GIII μ-conotoxins, the characterization of their action on Na_V_1.4 and their use in structure to function studies, nearly a decade and a half passed prior to the isolation of additional μ-conotoxin peptides. The timeline for the subsequent discovery of novel μ-conotoxins (also) displaying three inter-cysteine loops (CC---C---C---CC) and with C1-C4, C2-C5, and C3-C6 disulfide linkages, but with significant sequence diversity, is elegantly depicted in [90], and it resulted in the classification of the μ-conotoxin family as the M4 and M5 branches of the M-superfamily of *Conus* peptides (Table 2). Conotoxins without sequence similarity to the μ-conotoxins, and with four inter-cysteine loops (C---C---CC---C---C), were also isolated during that time [159] and classified in the O-superfamily as μO-conotoxins; their characterization on skeletal muscle and neuronal sodium channels is described later, along with a description of the characterization of ι-conotoxins with five inter-cysteine loops (C---C---CC---CC---C---C).

The isolation of PIIIA from *Conus purpurascens* [160] and its NMR solution structure [117] extended known μ-conotoxin targets to include neuronal voltage-gated sodium channels. PIIIA displayed a disulfide linkage similar to GIIIA, its competitive inhibition of STX binding for skeletal muscle was similar to GIIIA/B/C [117,173], and PIIIA Arg-14, homologous to the critical Arg-13 of *Conus geographus* μ-conotoxins, was an important determinant for inhibition of the skeletal muscle sodium channel. However, PIIIA was found to be a more competitive inhibitor than was GIIIA/B/C in rat or human brain. This peptide also blocked rat brain type II (Na_V_1.2) sodium channels with high affinity, unlike GIIIA, and displayed sequence divergence from GIIIA-C. These studies and investigations by [174,175] revealed several significant differences between GIIIA and PIIIA in their block of sodium channels. First, native PIIIA exhibited 10-fold less affinity for the Na_V_1.4 muscle channel compared to GIIIA. Second, alanine substitution produced more decrement of channel block for GIIIA R13-A compared to PIIIA R14-A, with approximately two-fold more residual current observed in the PIIIA mutant. Charge-altering substitutions of several residues in PIIIA also decreased the voltage dependence of channel block. In combination with molecular dynamics simulations, these results suggest that PIIIA R12, R20, R14, and K17 residues insert deep into the outer vestibule, with R14 and K17 entering into the transmembrane electric field and thus in proximity to the selectivity filter [174], consistent with the results from functional characterization and simulations of GIIIA/pore residue interactions [146,147]. Interestingly, a nonconserved histidine (H19) in PIIIA may also favor binding towards neuronal channels [175], as glutamine substitution at that residue increased PIIIA affinity towards Na_V_1.4. In GIIIA, that locus is a native glutamine, and GIIIA shows increased selectivity towards Na_V_1.4 with respect to PIIIA.

As neuronal sodium channel genes were cloned and associated with pathologies including epilepsy, familial hemiplegic migraine, and peripheral neuropathies, an important focus of the research on PIIIA and subsequently isolated μ-conotoxins was the determination of the affinities of native or mutant μ-conotoxin peptides to specific brain or peripheral nerve sodium channel isoforms. For example, soon after its isolation, PIIIA was shown to inhibit brain sodium channel Na_V_1.2 more effectively than PN1 of peripheral nerve [176], and this difference was utilized to track the developmental profile of brain versus peripheral nerve sodium channels in PC12 cells.

The GIIIA/B/C and PIIIA μ-conotoxins described above share a common pro-peptide sequence and cysteine framework and are classified in the M4 branch of the M superfamily of conotoxins [87,89,90]. The isolation of SmIIIA [164] extended the μ-conotoxins to a second, M5 branch of this superfamily. Functional characterization of SmIIIA actions on sensory (dorsal root ganglion) and autonomic (sympathetic chain ganglia) neurons revealed that this μ-conotoxin preferentially inhibited TTX-resistant sodium current, and that this action was not readily reversible. A chimeric approach was used [177] to investigate the locus for toxin affinity to TTX-resistant sodium channels in these neuronal populations, by swapping toxin regions between PIIIA, which does not affect TTX-resistant current, and SmIIIA, which does. Their results localized that site to residues in inter-cysteine loop 3 SmIIIA C-terminal half residues swapped into PIIIA conferred the toxin with ability to block TTX-resistant current, whereas the complementary swap of C-terminal half residues of PIIIA into SmIIIA abolished the capacity of that toxin to block TTX-resistant current.

Soon thereafter, two additional M5 conotoxins, KIIIA and SIIIA, were isolated and also found to inhibit the TTX-resistant sodium current in dorsal root and sympathetic ganglia [165]. Interestingly, while SmIIIA potently inhibited the compound action potential in frog skeletal muscle, this effect was not observed for KIIIA or SIIIA. Several differences in the sequences of the M5 conotoxins were noted as targets of investigation for the observed specificity of KIIIA and SIIIA towards neuronal sodium channels. KIIIA and SIIIA each exhibit a reduced number of residues in loop 1, the absence of heretofore conserved positively charged Arg or Lys residues in this loop, and the “substitution” at the critical Arg-13 position in loop 2 by lysine (R13 in GIIIA, PIIIA, and SmIIIA, but K7 for KIIIA, and K11 for SIIIA).

**Figure 5:**
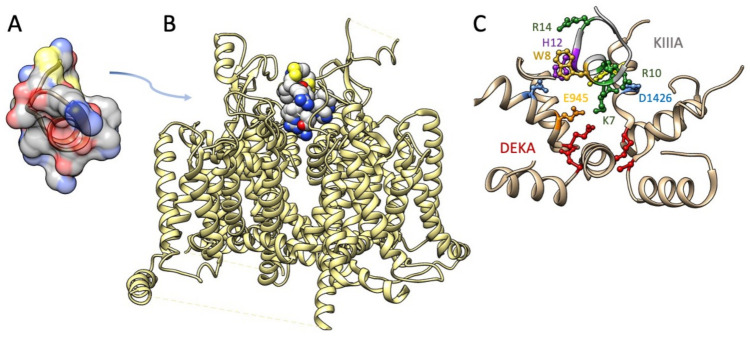
(**A**) Structure of KIIIA (.pdb 7SAV; [179]) (**B**) Cryo-EM structure of Na_V_1.2 bound by KIIIA (.pdb 6J8E; [152]). (**C**) Enlargement of the P-loops of Na_V_1.2 (tan helices) with KIIIA toxin residue K7 interacting with outer pore residues E945 and D1426, and R10 (shown in two possible orientations) interacting with D1426. Toxin residues W8, H12, and R14 are shown for which additional interactions within the P-loops, or with extracellular residues, were noted. DEKA selectivity filter residues are shown in red.

The M5 conotoxin KIIIA interaction with Na_V_1.2 has been determined in cryo-EM [152] (Figure 5). Several features of toxin interaction with its sodium channel target are captured in that structural determination. The critical Lys-7 (K7) in the helical portion of KIIIA extends deep into the pore towards the DEKA selectivity filter, similar to the poses proposed for Arg-13 from functional and computational investigations with GIIIA. K7 and other positively charged toxin residues including R10 interact with outer pore residues above the selectivity filter. Additional toxin residues including aromatic residues W8 and H12 interact with other pore and extracellular residues. This structural investigation supported a mode of μ-conotoxin binding in general as comprising electrostatic, polar, and H-bond interactions of positive residues on one face of the toxin with pore and other residues, and occlusion of the pore by KIIIA distinguished from that by TTX or STX with the KIIIA binding pose extending higher from the selectivity filter to occupy the outer pore vestibule. These differential binding poses are also supported by functional experiments showing that KIIIA could trap TTX or STX in its position lower in the pore and resulting in a slower rate of dissociation [178].

Functional characterization of μ-conotoxins with bioassays continued with the discovery of additional peptides in the M superfamily. Four M5 μ-conotoxins (CIIIA from *Conus catus*, CnIIIA, CnIIIB from *Conus consors*, and MIIIA from *Conus magus* were isolated and characterized [167]. CIIIA and CnIIIA blocked the TTX-resistant sodium current in dorsal root ganglion neurons with potency similar to KIIIA whereas SIIIA, MIIIA, and CnIIIB showed reduced potency. Interestingly, CIIIA was nonselective, as it blocked skeletal muscle action potentials, reminiscent of the profile exhibited by SmIIIA. CnIIIC was isolated [171] and exhibited similar potency on skeletal muscle contraction compared to TIIIA, and it inhibited the compound action potential elicited in myelinated and unmyelinated nerve preparations; inhibition of nerves by CnIIIC was not readily reversible. As additional M4 and M5 μ-conotoxins were isolated, their functional characterization typically utilized heterologous expression systems to test for isoform specificity of toxin action, as described below.

The isolation of μ-conotoxins other than GIIIA/B/C and PIIIA in the M4 branch, and the discovery and isolation of M5 branch μ-conotoxins, provided a wealth of native μ-conotoxins as potential specific antagonists of sodium permeance of sodium channel isoforms in the central and peripheral nervous system. The development of heterologous expression systems (*Xenopus* oocytes and mammalian embryonic cells) and incorporation of sodium channel genes into bacterial plasmids for expression of those sodium channels provided an important approach to determine the specificity of μ-conotoxins. Sodium channel nomenclature for isoforms was established [180], with Na_V_1.1, Na_V_1.2, and Na_V_1.3 found in brain; Na_V_1.4 in skeletal muscle; Na_V_1.5 in cardiac muscle; Na_V_1.6 in brain including the cerebellum, and peripheral nerve; and Na_V_1.7, Na_V_1.8, and Na_V_1.9 in peripheral nerve and including their relative sensitivity to TTX (Na_V_ 1.1–1.4, 1.6, 1.7) or resistance to TTX (Na_V_1.5, 1.8, 1.9). Together with the cloning and functional expression of each of the sodium channel isoforms in muscle, brain, and peripheral nerve, an important focus of investigation was to establish the pharmacological profile of novel conotoxins across neuronal, skeletal, and cardiac muscle sodium channels. These studies supported previous radiolabeling competition studies of the effects of μ-conotoxins on brain and muscle tissues, as well as bioassays of muscle and nerve preparations. In addition, they provided a reductionist determination of the relative affinities of M4 and M5 μ-conotoxins for specific sodium channel isoforms in these tissues.

As the complement of isolated μ-conotoxins grew, their functional characterization revealed a diversity of target sodium channels. Development of the pharmacological profile of μ-conotoxin action on channels expressed in oocytes or in mammalian cells was needed to provide a path forward in the development of μ-conotoxin analogues for treatment of channelopathies of brain, muscle, and peripheral nerve (reviewed by [26]). For the M4 branch of μ-conotoxins, GIIIA exhibited a 100-fold increase in potency on Na_V_1.4 compared to its actions Na_V_1.2 (reviewed by [19,20]). Interestingly, GIIIB produced significant block of Na_V_1.3 [161]. Similar to GIIIA, PIIIA also blocked Na_V_1.4 with greater potency than that observed for Na_V_1.2, but to a lesser extent [176]. The M4 μ-conotoxin TIIIA isolated from *Conus tulipa* produced effects similar to PIIIA, with significant but lesser block of NaV1.2 compared to that of Na_V_1.4 [161], and it was without action on Na_V_1.3, Na_V_1.5, Na_V_1.7, or Na_V_1.8 expressed in *Xenopus* oocytes. However, TIIIA did inhibit Na_V_1.7 endogenously expressed in neuroblastoma cells [181] at the relatively high dose of 1 μM. SxIIIA action was first assessed on Na_V_1.4, where it produced a rapidly reversible block [162] such as observed for GIIIA. The SxIIIA toxin was later shown to block Na_V_1.1, Nav1.6, and Na_V_1.2 with less potency compared to that for Na_V_1.4 [182]. More recently, the M4 μ-conotoxin TsIIIA was isolated from *Conus tessulatus* and found to inhibit TTX-resistant sodium current in dorsal root ganglion neurons [163]. When tested on sodium channels expressed in mammalian cells, TsIIIA had no effect on TTX-sensitive channels Na_V_1.1, Na_V_1.2, Na_V_1.3, Na_V_1.4, Na_V_1.6, or Na_V_1.7; it did not block TTX-resistant Na_V_1.5 but blocked TTX-resistant Na_V_1.8 with an IC_50_ at 2.1 μM [183]. 

The M5 conotoxin KIIIA blocked Na_V_1.2 to a slightly greater extent compared to Na_V_1.4 [184], and its block of Na_V_1.2 was not readily reversible, as is typically observed for the M4 branch. Alanine substitution of KIIIA Trp-8 (W8) resulted in a significant decrease in toxin block, and structural determination of KIIIA interaction with Na_V_1.2 revealed a strong interaction of W8 with DII extracellular Tyr-362 [152]; that aromatic residue is conserved in TTX-sensitive channels but not conserved in TTX-resistant channels Na_V_1.5 or Na_V_1.8. KIIIA partially blocked Na_V_1.1, Na_V_1.3, Na_V_1.6, and Na_V_1.7, extending the determination of neuronal isoforms targeted by μ-conotoxins [184,185]. The N-terminally extended isomer of KIIIA, KIIIB, was also shown to be a potent inhibitor of Na_V_1.2, with significant effects on Na_V_1.7 [185]. Certain isomers influencing disulfide connectivity of KIIIA had significant impact on its binding to Na_V_1.2, Na_V_1.4, and Na_V_1.7 [179]. The structural determination of SIIIA and the novel μ-conotoxin SIIIB revealed their sequence diversity, with an alpha helical motif conferring binding affinity for this toxin of equivalent potency on Na_V_1.2 and Na_V_1.4 [168]. That study, and [186], showed that both KIIIA and SIIIA blocked Na_V_1.2 irreversibly. Structural data on SIIIA in both studies suggested the importance of the C-terminal half of the peptide to promote neuronal sodium channel affinity; a later study concluded that N-terminal residues also contribute [187]. SIIIA was shown to act on other neuronal sodium channels [186,188] including Na_V_1.6 and to a lesser extent Na_V_1.7. The chimeric (Na_V_1.4 and Na_V_1.5 domain-swapping) and mutagenesis approaches utilized in [188] localized pore loop residues in domains I and II as structural determinants of SIIIA discrimination between sodium channel isoforms. While heterologously expressed TTX-resistant channels were not affected by KIIIA or SIIIA in these studies, each of these μ-conotoxins produced a persistent decrement of TTX-resistant sodium current in dorsal root ganglion neurons [165,166]. 

CnIIIC effectively and irreversibly blocked Na_V_1.4 expressed in mammalian cells [171,189]. Similar to SIIIA, its potency on Na_V_1.2 was similar to that of Na_V_1.4, with partial block of Na_V_1.6 and Na_V_1.7, with no effect on TTX-resistant sodium channels Na_V_1.5 and Na_V_1.8, and surprisingly with nanomolar block of the well-characterized α3β2 nicotinic acetylcholine receptor. BuIII A, BuIIIB, and BuIIIC isolated from *Conus bullatus* each blocked Na_V_1.4 to the same extent as M5 μ-conotoxin KIIIA [169]. Similar to the action of KIIIA, a BuIIIB block of Na_V_1.4 was not readily reversible. A subsequent study showed that BuIIIB also produced significant block of Na_V_1.1, Na_V_1.2, and Na_V_1.3 [182], with partial and lesser block for Na_V_1.5 and Na_V_1.6, respectively. 

## 6. Structural Divergence in M4 and M5 Conotoxins Affecting Channel Selectivity

The above studies on M5 μ-conotoxins extended the neuronal actions of conotoxins from Na_V_1.2 to several additional TTX-sensitive sodium channel isoforms. Shorter peptide analogues of BuIIIC and KIIIA suggested promise as candidates for increased selectivity towards block of neuronal sodium channels [190]. All of the conotoxins of the M5 branch possess a unique tryptophan residue in the third inter-cysteine loop, for which potential π/π and cation/π interactions with neighboring histidine and arginine residues were suggested as a possible structural determinant of the potency of KIIIA or SmIIIA interaction with neuronal channels [152,177,184,191]. These studies provided strong evidence that this aromatic residue is a determinant in isoform specific actions of M5 μ-conotoxins. Comparison of the actions of the M4 μ-conotoxin TIIIA isolated from *Conus tulipa* [161] to those of M5 μ-conotoxins SIIIA and SIIIB isolated from *Conus striatus* [168] provided additional insight into stable μ-conotoxin conformations and the important role of these homologous loci. SxIIIC was isolated from *Conus striolatus* [172], and its characterization revealed its block of several neuronal sodium channel isoforms including Na_V_1.3, Na_V_1.1, Na_V_1.6, Na_V_1.7, and Na_V_1.2 in order of potency. A subsequent comparison of relative potencies of μ-conotoxins on Na_V_1.7 showed that three μ-conotoxins with reported block of Na_V_1.7, SxIIIC, KIIIA, and SmIIIA blocked that sodium channel isoform with the greatest variability compared to actions on other neuronal channels [192]. That study explored structural differences in KIIIA, SmIIIA, and SxIIIC, focused on N-terminal residue extension in SmIIIA and SxIIIC compared to KIIIA, number of residues in loop 1, and charged residues in loop 3. Their findings revealed that number of loop 1 residues are a determinant in the relative potencies of SmIIIA, SxIIIC, and KIIIA, and that charged residues in loop 3, as contributing to overall net charge of toxin surface of M4 *versus* M5 μ-conotoxins, are an important factor in selectivity for Na_V_1.7.

The aforementioned studies showed that μ-conotoxins of the M superfamily have affinity to the outer vestibule of the pore module in voltage-gated sodium channels, with select residues extending to the selectivity filter, with pore-blocking actions such as those of saxitoxin and tetrodotoxin, through their interactions with binding sites overlapping with those of marine toxins in receptor site 1. The initial characterization of *Conus geographus* μ-conotoxins determined critical interaction between toxin residues and those of the skeletal muscle sodium channel pore. Subsequent, isolation, structural determination and functional characterization of additional M4 and M5 μ-conotoxins shown in Table 2 revealed a diversity of toxin sequences and neuronal sodium channel isoforms targeted, enhancing our understanding of toxin action and expanding the possibilities for development of isoform specific μ-conotoxin analogues to investigate neuropathic sodium channelopathies, and provide alternative intervention in these diseases.

## 7. Isolation and Characterization of μO-Conotoxins

As μ-conotoxins were being isolated and studied, μO- and δ-conotoxins of the O superfamily were also discovered and functionally characterized for their actions on voltage-gated sodium channels (Table 3). μO-conotoxins were isolated from *Conus marmoreus* as MrVIA and MrVIB [159].

These peptides exhibit sequence similarity to δ-conotoxins (targeting sodium channels), ω-conotoxins (targeting calcium channels), and κ-conotoxins (targeting potassium channels). Their cysteine framework is distinguished from that of the μ-conotoxins by a disulfide cysteine pattern within the peptide, named the inhibitory cysteine knot motif (ICK), with the terminal disulfide bridge passing through the polypeptide backbone and imparting a compact globular structure [193]. This pattern has been identified as a common structural determinant of peptide toxins acting on a diversity of ion channels (reviewed by [194,195]) and comprises four inter-cysteine loops (C---C---CC---C---C), compared to three such loops as determined for μ-conotoxins (Table 1). MrVIA and MrVIB were thus classified as μO-conotoxins with their general tertiary structure as defined by their polypeptide precursor sequence, the resulting cysteine knot pattern and with their ability to inhibit sodium current.

**Table 3:** Sequences of μO- and δ-conotoxin gating modifiers of voltage-gated sodium channels. Sequences characterized with cysteine framework ---C---C---CC---C---C---.

Toxin	*Conus* Species	Sequence	References
μ-O		--- ** C ** ------ ** C ** ------------- ** CC ** ---- ** C ** ---- ** C ** ---	
MrVIA	*C. marmoreus*	--A ** C ** RKKWEY ** C ** ----IVPIIGFIY ** CC ** PGLI ** C ** GPFV ** C ** V----	[159]
MrVIB	*C. marmoreus*	--A ** C ** SKKWEY ** C ** ----IVPILGFVY ** CC ** PGLI ** C ** GPFV ** C ** V----	[159]
MfVIA	*C. magnificus*	-RD ** C ** QEKWEY ** C ** ----IVPILGFVY ** CC ** PGLI ** C ** GPFV ** C ** V----	[196]
δ			
Tx(V)IA	*C. textile*	--W ** C ** KQSGEM ** C ** N---LLD--QN-- ** CC ** DGY- ** C ** IVLV ** C ** T----	[197,198,199]
TxIB	*C. textile*	--W ** C ** KQSGEM ** C ** N---VLD--QN-- ** CC ** DGY- ** C ** IVFV ** C ** T----	[198]
GmVIA	*C. gloriamus*	VKP ** C ** RKEGQL ** C ** D-----PIFQN-- ** CC ** RGWN ** C ** V-LF ** C ** V----	[200]
NgVIA	*C. nigropunctatus*	-SK ** C ** FSOGTF ** C ** G---IKO--GL-- ** CC ** SVR- ** C ** FSLF ** C ** ISFE-	[201]
PVIA	*C. purpurascens*	-EA ** C ** YAOGTF ** C ** G---IKO--GL-- ** CC ** SEF- ** C ** LPGV ** C ** FG---	[202]
SVIE	*C. striatus*	-DG ** C ** SSGGTF ** C ** G---IHO--GL-- ** CC ** SEF- ** C ** F-LW ** C ** ITFID	[203]
CnVIA	*C. consors*	-YE ** C ** YSTGTF ** C ** G---ING--GL-- ** CC ** SNL- ** C ** LFFV ** C ** LTFS	[203]
Am2766	*C. amadis*	--- ** C ** KQAGES ** C ** D---IFS--QN-- ** CC ** VGT- ** C ** A-FI ** C ** IE---	[204]
EVIA	*C. ermineus*	-DD ** C ** IKOYGF ** C ** SLPILKN--GL-- ** CC ** SGA- ** C ** V-GV ** C ** ADL--	[205]
CnVIB	*C. consors*	-DE ** C ** FSOGTF ** C ** G---TKO--GL-- ** CC ** SAR- ** C ** FSFF ** C ** ISLEF	[206]
CnVIC	*C. consors*	-DE ** C ** FSOGTF ** C ** G---IKO--GL-- ** CC ** SAR- ** C ** LSFF ** C ** ISLEF	[206]
CnVID	*C. consors*	-DE ** C ** FSOGTF ** C ** G---FKO--GL-- ** CC ** SAR- ** C ** FSLF ** C ** ISLEF	[206]
TsVIA	*C. tessulatus*	--- ** C ** AAFGSF ** C ** G---L-P--GLVD ** CC ** SGR- ** C ** F-IV ** C ** LL---	[207]
SuVIA	*C. suturatus*	--- ** C ** AGIGSF ** C ** G---L-P--GLVD ** CC ** SDR- ** C ** F-IV ** C ** LP---	[2]

MrVIA blocked sodium current in molluscan (*Aplysia*) neurons and produced ataxia and/or coma when injected at nanomole doses (or less) into the central nervous system (intra-cranial injection) of mice [159]. Interestingly, MrVIA was without effect when injected into the periphery and thus accessible to muscle (inter-peritoneal injection). In contrast, the effects of μ-conotoxin GIIIB are observed for peripheral, but not central, injections [99]. The μO-conotoxins were of considerable interest for their apparent neuronal specificity, as noted several years before the discovery of M5 μ-conotoxins acting on voltage-gated sodium channels in brain and peripheral nerve. MrVIA inhibited TTX-sensitive sodium currents in cultured hippocampal neurons [208], and Na_V_1.2 expressed in *Xenopus* oocytes with nanomolar potency. This effect on Na_V_1.2 was the result of a hyperpolarized shift of the steady-state fast inactivation curve, suggesting its action was not to block permeation as for GIIIA. Indeed, MrVIA did not displace STX from brain or electric eel tissue in radiolabel binding assay, suggesting the toxin does not inhibit sodium channels through interaction with receptor site 1.

Receptor site 4 is targeted by β-scorpion toxins [209,210] and the inhibitory cysteine knot tarantula toxin ProTx-II [211]. This site includes residues of domain II S1–S2 and S3–S4 extracellular loops [212,213,214] and extends to the N-terminal pore loop in domain III, proximal to the DII VSD. β-scorpion and spider toxins act as gating modifiers by “trapping” the voltage sensor in domain II in the activated position (for reviews of voltage sensor trapping see [76,77]; Figure 1C,D). MrVIA inhibited Na_V_1.4 DII voltage sensor translocation [215], and a chimeric approach revealed its interaction with the N-terminal pore loop in domain III [216]. These findings suggested overlapping binding sites for MrVIA, β-scorpion, and spider toxins. MrVIA trapping of the domain II voltage sensor in Na_V_1.4 was effectively competed against by β-scorpion toxin, which did not compete against pore block by GIIIA [215]. MrVIA, but not GIIIA inhibition of Na_V_1.4 was relieved by repetitive depolarization, an action also observed for the β-scorpion toxins acting on receptor site 4. Thus, the μO-conotoxins prohibit sodium permeance as a consequence of their effect on DII voltage sensor translocation rather than through pore occlusion, as observed for the μ-conotoxins.

Attention was focused on the specificity of MrVIA and MrVIB μO-conotoxins for neuronal sodium channel isoforms and its potential use as a pharmacological intervention in peripheral neuropathies associated with TTX-resistant sodium channels Na_V_1.8 and Na_V_1.9. Both MrVIA [217] and MrVIB [218] inhibited action potentials in nociceptive C fibers and produced analgesic effects in pain assays in mice. MrVIA was shown to be more effective at inhibiting TTX-resistant versus TTX-sensitive sodium current in dorsal root ganglion neurons [219], similar to the actions of μ-conotoxins SMIIIA [164] and KIIIA [165]. MrVIB also blocked TTX-resistant sodium current in these neurons, and it specifically inhibited Na_V_1.8 in heterologous expression [218]. Recovery of Na_V_1.8 current amplitude was prolonged, unless strong depolarization was used to condition the channels during that recovery, presumably due to displacement of the toxin from receptor site 4 with voltage sensor translocation. Interestingly, rate of MrVIB block was accelerated by the presence of any of the β 1–4 subunits co-expressed with the α subunit of the sodium channel [220]. A similar effect was subsequently noted for β subunit modulation of the rate of pore block of Na_V_1.1, Na_V_1.2, Na_V_1.6, or Na_V_1.7 by μ-conotoxins TIIIA, PIIIA, SmIIIA, and KIIIA [221]. Thus, while STX block of the pore is insensitive to β subunit presence [220], β subunits do modulate the kinetics of sodium channel inhibition mediated either by μ-conotoxin pore blockers or μO-conotoxin gating modifiers.

The related μO-conotoxin MfVIA was isolated from *Conus magnificus* [196]. This conotoxin, similar to MrVIA and MrVIB, blocked TTX-resistant sodium current in dorsal root ganglion neurons, as well as TTX-sensitive Na_V_1.4 and TTX-resistant Na_V_1.8 sodium channels in heterologous expression. The study by [196] also noted the increased potency of these μO-conotoxins on TTX-resistant current in neurons compared to that expressed in oocytes or mammalian cells, hypothesizing that this difference could be due to the demonstrated action of β subunits on the rate of block, as shown for MrVIB [220]. MfVIA μO-conotoxin was used in a study combining mutagenesis and automated patch clamp technology [93]. Here, the double mutant MrVIA E5K/E8K produced a significant increase in potency and provided analgesia in a formalin-based behavioral assay for pain. Two additional μO-conotoxins were isolated from *Conus litteratus* [222,223], each of which inhibit sodium current in dorsal root ganglion neurons but have not yet been tested in heterologous expression for isoform specificity.

## 8. Delta (δ-)Conotoxins

Delta (δ)-conotoxins share sequence similarity to the ω-conotoxins, κ-conotoxins, and μO-conotoxins but not the μ-conotoxins (reviewed by [81,224]; Table 3), with the C---C---CC---C---C--- cysteine framework and four inter-cysteine loops and considerable hydrophobicity of amino acid residues of those loops, in contrast to the μ-conotoxins ([94]. For mammalian sodium channel isoforms targeted, the impact of δ-conotoxins was observed as a pronounced slowing of the entry of channels into a state of fast inactivation, similar to the action of α-scorpion (reviewed by [225]) or sea anemone toxins (reviewed by [58,59]; Figure 3). The S4 segment in domain IV acts as the voltage sensor for fast inactivation [226], and toxins acting at receptor site-3 trap the domain IV S4 segment in a resting or intermediate configuration ([119,227,228]; Figure 1D).

Several investigations of the effect of δ-conotoxins on sodium current or action potentials in molluscan neurons [198,200,229,230] preceded electrophysiological characterization using mammalian sodium channels in heterologous expression. Am2766 from *Conus amadis* [204,231] slowed the entry of Na_V_1.2 into the fast-inactivated state; the related peptide Am2755 differs from Am2766 in the C-terminus and has no effect on Na_V_1.2 fast inactivation. EVIA (*Conus ermineus*) prolonged fast inactivation of Na_V_1.2, Na_V_1.3, and Na_V_1.6 sodium channels without affecting Na_V_1.4 or Na_V_1.5 [205]. SVIE (*Conus striatus*), first characterized by its effect to slow sodium current inactivation in frog sympathetic ganglion neurons [203,232], was later employed in studies to define the binding site. Alpha scorpion toxin Lqh-2 and alpha-like toxin Lqh-3 have overlapping but distinct site-3 binding sites in the domain IV S3–S4 linker [233]. A comparison of sodium channel Na_V_1.4 response to Lqh-2, Lqh-3, and SVIE, and incorporating cysteine scanning mutagenesis of the S3–S4 linker, revealed an important hydrophobic interaction of the δ-conotoxin with a conserved YFV motif as part of the receptor 6 binding site for δ-conotoxins [80]. A comparison of the kinetic block and gating modifying actions of μO- and δ-conotoxins is provided in the review by [81]. 

CnVIB, CnVIC and CnVID (*Conus consors*) slowed fast inactivation in several TTX-sensitive channels, with effects pronounced for CnVIC/D on Na_V_1.2, CnVID on Na_V_1.3, CnVIB/C on Na_V_1.4, and each prohibiting fast inactivation of Na_V_1.6 [206]. While TTX-resistant channels Na_V_1.5 and Na_V_1.8 were largely unaffected, CnVIC did elicit a slight slowing of fast inactivation in Na_V_1.5, illustrating significant sodium channel isoform specificity for these δ-conotoxins. TsVIA (*Conus tessulatus*) enhanced the depolarizing influence of the site-2 toxin veratridine and slowed fast inactivation of Na_V_1.6 [207]. SuVIA (*Conus suturatus*) was shown to enhance veratridine depolarization for Na_V_1.3, Na_V_1.4, Na_V_1.6, and Na_V_1.7, using a fluorescence-based membrane potential assay [2]. In whole cell recordings, 5 nM SuVIA produced an (excitatory) left shift of the conductance curve for on Na_V_1.7, without significant effect on fast inactivation kinetics. Thus, δ-conotoxins typically act as gating modifiers with toxin specificity towards TTX-sensitive channels, promoting their excitatory effect by slowing the entry of sodium channels into the fast-inactivated state or enhancing activation.

## 9. Isolation and Characterization of Iota (ι)-Conotoxins

Conotoxins of the I superfamily exhibit a structure characterized by eight cysteine residues as C---C---CC---CC---C---C with five inter-cysteine loops [234,235]. Of five similar “iota-conotoxin” peptides in this superfamily purified from *Conus radiatus*, r11a, r11b, r11c, and r11e elicited repetitive action potentials in nerve or muscle when applied to the frog cutaneous pectoris neuromuscular preparation. These and other I-1 conotoxins isolated from *Conus figulinus*, *Conus magus*, and *Conus striatus* were isolated, and posttranslational modification of r11a was shown to increase its biological activity [236].

The first of these I-1 conotoxins characterized on mammalian sodium channels was ι-RXIA (r11a), which increased current amplitude and produced an excitatory left shift of the activation probability curve for Na_V_1.6 expressed in *Xenopus* oocytes [95] without affecting steady-state fast inactivation. ι-RXIA was shown to produce similar effects on Na_V_1.2 and Na_V_1.7, at higher doses [237], and it promoted increased action potential frequency in A (proprioceptive) fibers known to express Na_V_1.6, and C (nociceptive) fibers known to express Na_V_1.7. Thus, both the δ- and ι-conotoxins are excitatory in their effect, albeit typically acting on different gating transitions of voltage-gated sodium channels.

## 10. Summary

In conclusion, conotoxins have provided unique opportunities to study the structure-to-function relationships of voltage-gated sodium channels, within the context of an intriguing evolutionary background of considerable interest to many in the field of toxin biology. In addition to the conotoxins of the M, O, and I superfamilies characterized by functional experiments described in this review, other conotoxins appear to target voltage-gated sodium channels. For example, conotoxin GS was isolated, with a sequence exhibiting six cysteine residues and with ability to displace TTX from skeletal muscle homogenate [238]. The conotoxin TvIIA isolated from *Conus tulipa* displays structural similarities to μO-conotoxins, δ-conotoxins, and to GS conotoxin, but has not been tested for actions on sodium channels [239]. A four cysteine conotoxin from the T superfamily, sr5a, was isolated from *Conus spurius* [240]. This conotoxin was subsequently tested on expressed sodium channels and found to produce partial block of TTX-resistant Na_V_1.5 at 0.2 μM, with lesser block of TTX-sensitive Na_V_1.6 or Na_V_1.7 [241] and named μ-SrVa. These studies illustrate the importance of continued discovery and characterization of novel conotoxins targeting voltage-gated sodium channels.

A few of the other approaches and questions that have shared this historical path towards discovery should be noted, albeit in brief. Computational approaches such as docking studies using homology models, or molecular dynamics, have been instrumental in understanding sodium channel function and their interactions with conotoxins [165,242,243]; for reviews see [24,244,245], including prokaryotic sodium channels as part of this effort [246,247,248,249] and extending to examine the roles of toxin residues [250] or disulfide linkage connectivity on μ-conotoxin binding and block of sodium permeance [179,251,252].

Proteome and transcriptome analyses have played an increasingly important role in conotoxin research. The venomics approach has revealed numerous and interesting details with respect to the diversity, plasticity, post-translational modification, and evolution of conopeptides (for reviews see [253,254,255,256,257]). Along with computational approaches, these larger scale analyses provide opportunities for success in future investigations employing structural and functional characterization of these peptides. Finally, targeting post-translational modification with existing and new technologies may promote more rapid development of conotoxin analogues suitable for specific pharmacological intervention (reviewed by [258,259,260]) including new strategies for antimicrobials [261,262,263].

## Figures and Tables

**Table 1:** Conotoxins targeting voltage-gated sodium channels. Structural features are listed for cysteine framework, number of inter-cysteine loops, di-sulfide cysteine connectivity, fold pattern, and distinguishing motif; ICK is the inhibitory cysteine knot motif.

Superfamily	Conotoxins	Cysteine Pattern	Loops	Connectivity	Fold Pattern	Motif
M	μ-conotoxin	CC---C---C---CC	3	1–4, 2–5, 3–6	B	-
O	μO-conotoxin	C---C---CC---C---C	4	1–4, 2–5, 3–6	C	ICK
O	δ-conotoxin	C---C---CC---C---C	4	1–4, 2–5, 3–6	C	ICK
I	ι-conotoxin	C---C---CC---CC---C---C	5	1–4, 2–5, 3–6, 5–8	C	ICK

**Table 2:** Sequence alignments of μ-conotoxins blocking permeance in voltage-gated sodium channels. M4 and M5 branches distinguished in part by number of residues in inter-cysteine loop 3. O = hydroxyproline, Z = pyroglutamate.

Toxin	*Conus* Species	Sequence	Reference
M4 branch		--- ** CC ** ------ ** C ** ---- ** C ** ---- ** CC ** ---	
GIIIA	*C. geographus*	-RD ** CC ** TOOKK- ** C ** KDRQ ** C ** KOQR ** CC ** A-	[97,98,99]
GIIIB	*C. geographus*	-RD ** CC ** TOORK- ** C ** KDRR ** C ** KOMK ** CC ** A-	[97,98,99]
GIIIC	*C. geographus*	-RD ** CC ** TOOKK- ** C ** KDRR ** C ** KOLK ** CC ** A-	[99]
PIIIA	*C. purpurascens*	ZRL ** CC ** GFOKS- ** C ** RSRQ ** C ** KOHR ** CC ** -	[160]
TIIIA	*C. tulipa*	RHG ** CC ** KGOKG- ** C ** SSRE ** C ** ROQH ** CC ** -	[161]
SxIIIA	*C. striolatus*	--R ** CC ** TGKKGS ** C ** SGRA ** C ** KNLK ** CC ** A-	[162]
SxIIIB	*C. striolatus*	-QK ** CC ** TGKKGS ** C ** SGRA ** C ** KNLR ** CC ** A-	[162]
TsIIIA	*C. tessulatus*	--G ** CC ** RWP--- ** C ** PSR- ** C ** GMAR ** CC ** SS	[163]
M5 branch		---- ** CC ** -------- ** C ** ---- ** C ** ----- ** CC ** ---	
SmIIIA	*C. stercusmuscarum*	--ZR ** CC ** N---GRRG ** C ** SSRW ** C ** RDHSR ** CC ** ---	[164]
KIIIA	*C. kinoshitai*	---- ** CC ** N------- ** C ** SSKW ** C ** RDHSR ** CC ** ---	[165]
SIIIA	*C. striatus*	--ZN ** CC ** N---G--G ** C ** SSKW ** C ** RDHAR ** CC ** ---	[165,166]
CIIIA	*C. catus*	--GR ** CC ** E---GPNG ** C ** SSRW ** C ** KDHAR ** CC ** ---	[167]
CnIIIA	*C. consors*	--GR ** CC ** D---VPNA ** C ** SGRW ** C ** RDHAQ ** CC ** ---	[167]
CnIIIB	*C. consors*	--ZG ** CC ** G---EPNL ** C ** FTRW ** C ** RNNAR ** CC ** RQQ	[167]
MIIIA	*C. magus*	--ZG ** CC ** N---VPNG ** C ** SGRW ** C ** RDHAQ ** CC ** ---	[167]
SIIIB	*C. striatus*	--ZN ** CC ** N-----GG ** C ** SSKW ** C ** KGHAR ** CC ** ---	[168]
BuIIIA	*C. bullatus*	VTDR ** CC ** K---GKRE ** C ** -GRW ** C ** RDHSR ** CC ** ---	[169]
BuIIIB	*C. bullatus*	VGER ** CC ** K--NGKRG ** C ** -GRW ** C ** RDHSR ** CC ** ---	[169]
BuIIIC	*C. bullatus*	IVDR ** CC ** NKGNGKRG ** C ** -SRW ** C ** RDHSR ** CC ** ---	[169]
KIIIB	*C. kinoshitai*	--NG ** CC ** N------- ** C ** SSKW ** C ** RDHSR ** CC ** ---	[170]
CnIIIC	*C. consors*	--ZG ** CC ** N---GPKG ** C ** SSKW ** C ** RDHAR ** CC ** ---	[171]
SxIIIC	*C. striolatus*	--RG ** CC ** N---GRGG ** C ** SSRW ** C ** RDHAR ** CC ** ---	[172]

## Data Availability

Not applicable.
